# A qualitative systematic review of patients’ experience of osteoporosis using meta-ethnography

**DOI:** 10.1007/s11657-016-0286-z

**Published:** 2016-10-13

**Authors:** K. L. Barker, F. Toye, C. J. Minns Lowe

**Affiliations:** 1 Physiotherapy Research Unit, Nuffield Orthopaedic Centre, Oxford University Hospitals NHS Foundation Trust, Windmill Road, Oxford, OX3 7HE UK; 2 Nuffield Department of Orthopaedics, Rheumatology and Musculoskeletal Sciences, University of Oxford, Oxford, OX3 7LD UK

**Keywords:** Qualitative research, Osteoporosis, Osteopenia, Patient experience, Systematic review, Meta-ethnography

## Abstract

***Summary*:**

We aimed to systematically review qualitative studies exploring the experience of living with osteoporosis to develop new conceptual understanding. We identified themes about the invisibility/visibility of osteoporosis, the experience of uncertainty of living with osteoporosis (OP) and living with an ageing body and the place of gender.

**Purpose:**

The aim of this review was to systematically review the body of qualitative studies exploring the experience of living with either osteoporosis or osteopenia and to use meta-ethnography to develop new conceptual understanding.

**Methods:**

We systematically reviewed and integrated the findings of qualitative research from four bibliographic databases (Medline, Embase, Cinahl, Psychinfo) to September 2015 in order to increase our conceptual understanding of the lived experience of osteoporosis and osteopenia. Articles were appraised for quality; each was independently read by two researchers to identify concepts which were compared and developed into a conceptual model.

**Results:**

Our findings demonstrate that coming to terms with a diagnosis of osteoporosis is linked to its relative visibility or invisibility. For some, OP has not become manifest and self-identity is intact (biographical integrity). For others, OP is profoundly manifest and self-identity is no long intact (biographical fracture). We also demonstrate that overwhelming uncertainty pervades the experience of OP. Our final theme demonstrates how the experience of OP is set within a cultural context with certain views about ageing and gender.

**Conclusions:**

Our synthesis has highlighted the wealth of qualitative data about osteoporosis and osteopenia. Despite the increasing body of literature on the subject, there remains a need to adjust our interactions with patients. This will allow clinicians to understand how patients can be helped to receive and understand their diagnosis and move forward in partnership with healthcare providers to promote optimal management of the disease.

## Introduction

Osteoporosis (OP) is a global health issue with 1 in 3 women and 1 in 5 men over the age of 50 years, predicted to break a bone as a result of OP [1]. OP is one of the most common long-term conditions which can have a major impact on an individual’s health-related quality of life (QoL) due to pain, limitations in activity, social participation and altered mood [2, 3]. Qualitative research can help us to understand people’s experience of living with particular health conditions and allow us to contextualise the decisions that people make regarding their own health care. However, the proliferation of research and issues related to retrieving qualitative studies can make it difficult to use this knowledge [4]. Insights from qualitative syntheses have contributed to our understanding of complex processes such as medicine taking [5], adherence to diabetes treatments [6], use of antidepressants [7] and patients’ experience of chronic musculoskeletal pain [8] and pelvic pain [9]. The review was set within the context of an ongoing OP randomised controlled trial. This trial incorporates a qualitative study to explore the experiences and views of people with OP and vertebral fracture regarding the trial interventions, their perceptions regarding the appropriateness and acceptability of the interventions and to explore the factors influencing their adherence to the intervention programmes. There are various methods for synthesising qualitative research [10–12]. Studies range from those aiming to describe qualitative findings to studies that are more interpretive and generate theory. Meta-ethnography is an interpretive form of knowledge synthesis, proposed by Noblit and Hare [13], which aims to develop new conceptual understandings. The aim of this review was to systematically review the body of qualitative studies exploring the experience of living with OP to bring together the findings of published qualitative research.

## Method

### Meta-ethnography

Noblit and Hare [13] propose seven stages to a meta-ethnography synthesis which take the researcher from formulating a research idea to expressing the findings of research. These stages are not discrete but form part of an iterative research process. (1) *Getting started* involves formulating a research idea that is ‘worthy of the synthesis effort’. (2) *Deciding what is relevant* involves determining your search and inclusion strategy. (3) *Reading the studies* involves careful attention to the conceptual content of the included studies. (4) *Determining how studies are related* involves identifying and describing the ‘metaphors’ or concepts in studies and ‘translating’ or comparing them to those in other studies. This is fundamental to meta-ethnography where concepts are the raw data of the synthesis. (5) *Translating studies into each other* involves sorting concepts from primary studies into conceptual categories or ‘piles’, thus ‘translating qualitative studies into one another’. Translation is achieved through the constant comparative method [14]. (6) *Synthesising translations* involves developing a model that helps to make sense of the topic under investigation. (7) *Expressing the synthesis* involves output and dissemination of findings. This may differ from other synthesis approaches that stop analysis at the stage where they have theoretically saturated categories.

We included reports of qualitative studies that explored adults’ own experience of OP. Studies were included if participants had a diagnosis of OP or osteopenia, osteoporotic fracture or were taking medication for the treatment of OP. We searched four electronic bibliographic databases from inception until September 2015: Medline, Embase, Cinahl, Psychinfo. An example of search syntax is shown in Table 1. As meta-ethnography relies on identifying and defining concepts within each study, we chose to limit the search to English language. We used a combination of free text terms and thesaurus or subject headings. We refined search terms specific to qualitative research available from the InterTASC Information Specialists’ Sub-Group (ISSG) Search Filter Resource (www.york.ac.uk/inst/crd/intertasc/). We screened titles, abstracts or full texts to exclude articles that did not meet the inclusion criteria.

**Table 1:** Example search syntax

1. EMBASE; exp. OSTEOPOROSIS/; 95,733 results.
2. EMBASE; exp. QUALITATIVE RESEARCH/; 30,962 results.
3. EMBASE; (qualitative ADJ research).ti,ab; 8273 results.
4. EMBASE; (grounded ADJ theory).ti,ab; 7894 results.
5. EMBASE; NURSING METHODOLOGY RESEARCH/; 14,146 results.
6. EMBASE; exp. OSTEOPOROTIC FRACTURES/; 10,767 results.
7. EMBASE; ethnograph*.ti,ab; 7259 results.
8. EMBASE; phenomenol*.ti,ab; 18,506 results.
9. EMBASE; osteopen*.ti,ab; 12,435 results.
11. EMBASE; osteoporo*.ti,ab; 78,877 results.
12. EMBASE; 1 or 6 or 9 or 11; 119,566 results.
13. EMBASE; exp. ETHNOGRAPHY/ OR exp. ETHNOGRAPHIC RESEARCH/; 1872 results.
14. EMBASE; exp. PHENOMENOLOGY/; 7373 results.
15. EMBASE; exp. GROUNDED THEORY/; 2434 results.
16. EMBASE; 2 OR 3 OR 4 OR 5 OR 7 OR 8 OR 13 OR 14 OR 15; 75,721 results.
17. EMBASE; 12 AND 16; 134 results.

The use of quality criteria to determine inclusion for syntheses of qualitative studies has been challenged [15–19]. We know that quality appraisal does not produce consistent judgements [17]. The decision to appraise, or not, is confounded by the prevailing research culture where appraisal for qualitative synthesis is the expectation. It may be argued that excluding studies on the basis of quality criteria may exclude insightful studies [10]. Others argue that there may be a positive relationship between sound method and positive contribution to the synthesis [20]. We agreed that papers should provide an adequate methodological report [19]. KB and CML appraised all papers using the Critical Appraisal Skills Programme (CASP) for appraising qualitative research [21] as a focus for discussion on methodological adequacy. However, as central feature of meta-ethnography is that the *data* are the concepts [13] to be utilised within a meta-ethnography, studies must above all provide adequate description of their concepts [13].

We uploaded a full copy of all papers onto Nvivo 9 software to help organise the qualitative analysis [22]. NVivo 9 allows the collection, organisation and analysis of a large body of knowledge by. It also allows tracking of developing ideas and theories through ‘memos’.

We used the methods of meta-ethnography [13] to synthesise the data [10, 23, 24]. Central to meta-ethnography is identifying key ideas or ‘concepts’ and comparing these concepts across studies [13]. Two members of the team (FT and CML) read each paper to identify and describe the concepts. We compared these independent descriptions and developed a collaborative description. Our aim was not to reach consensus but to dialectically develop ideas. These refined concepts formed the primary data for the meta-ethnography. We did not re-organise or recode primary findings. If there was no clear concept articulated in the original study, then we labelled it *untranslatable* [19]. In short, if the original study was purely descriptive and needed recoding to decipher a clear idea, then there was no ‘data’ to analyse. FT and KB organised concepts into categories with shared meaning through constant comparison. FT developed a draft conceptual model [13] to draw the themes together into a framework and discussed and refined this model in collaboration with the team.

In order to ensure that we had incorporated the perspective of patients and service users into our analysis and review, we sought the input of a group of current service users who were attending an exercise group for OP to read, and then discuss with us, the conceptual categories as described in Table 2. Four patients agreed (two men and two women), and their input was incorporated into our overall findings.

**Table 2:** Conceptual categories and studies supporting

Descriptions of conceptual category discussed with patient user group		
Conceptual category	Biographical integrity—osteoporosis is not manifest	Study supporting concept
I know I have it but I can’t see it	My OP is not a problem. I have made a few changes so that I can still do the things that are important to me. Surely, pain would warn me if something was happening?	Besser [25]; de Souza [26]; Mazor [34]; Meadows [37]; Paier [40]; Richardson [43]; Sale [46]; Sale [47]; Solimeo [54]; Weston [55]
There was nothing fragile about it	My fracture was not because of weak bones. It was really traumatic. The doctor said anyone would have broken a bone if they had done that.	Beaton [23]; Besser [25]; Meadows [37]; Sale [46]; Sale [47]; Sale [50]
I am not the type to get osteoporosis	I know that OP can cause fractures, but I am not at risk because I have always had strong bones. I have a good diet and plenty of exercise. I am too young to get OP. I was therefore really shocked the scan was positive.	Mazor [34]; Meadows [37]; Richardson [43]; Roberto [44]; Sale [50]; Salter [53]
IT is not as bad as other conditions	It’s not as bad as having heart disease or diabetes. I mean, I could have lung cancer or dementia. I think I’m lucky.	French [27]; Jachna [32]; Mazor [34]; Salter [53]; Weston [55]
Biographical fracture—osteoporosis is manifest		
Osteoporosis choreographs my life	OP has disrupted my leisure, work, family and social life. It sometimes makes me feel angry, sad or frightened.	Hallrup [24]; Besser [25]; de Souza [26]; Hallberg [29]; Hansen [30]; Iversen [31]; Meadows [36]; Paier [40]; Roberto [44]; Salter [53]; Wilkins [56, 57]
I am becoming isolated	I sometimes feel isolated or lonely. I avoid social situations. It helps if I can share my experiences with other people. At times, I am dependent on my family and friends for social contact.	Hallrup [24]; Hallberg [29]; Nielson [38]; Qvist [42]; Wilkins [56, 57]
It is really difficult asking for help	Sometimes I need help and it is not always available. It is difficult asking for help (especially with personal care) as it makes me feel like I am getting old. I appreciate their help, but at times, my family can be a bit ‘too helpful’. I really don’t want to rely on my friends and family.	Hallrup [24]; Hallberg [29]; Jachna [32]; Roberto [44]; Salter [53]
Living in fear of falls	I am worried about doing activities that I never used to worry about. I might fall and fracture. I take a lot of care to not fall over. This sometimes means that I stay at home in a safe environment.	Beaton [23]; Hallrup [24]; Besser [25]; Giangregorio [28]; Hallberg [29]; Meadows [36]; Paier [40]; Sale [50]
Fear of what is to come	I don’t know what the future will bring. I am worried that I won’t be able to get about or keep my independence. I am worried about breaking another bone. I am worried about what I will look like.	Beaton [23]; Hallrup [24]; Besser [25]; Mazor [34]; Nielsen [38]; Paier [40]; Quantock [41]; Roberto [44]; Sale [50]; Wilkins [56]; Wilkins [57]
I am watching my body get old	I am starting to look like an old person way before my time. I used to know someone who was in marvellous shape before she got OP.	Hallrup [24]; Hallberg [29]; Hansen [30]; Iversen [31]; Paier [40]; Roberto [44]; Wilkins [56, 57]
Overwhelming uncertainty		
What is my risk?	I don’t know whether I am at risk of breaking a bone or not. Surely, everyone gets a bit of OP. My doctor has not told me about my test results. I suppose no news is good news. I hear different things from different people. I try and find out as much as I can.	Beaton [23]; Hallrup [29]; Besser [25]; French [27]; Giangregorio [28]; Hansen [30]; Iversen [31]; Lau [33] Mazor [34]; Meadows [37]; Paier [40]; Quantock [41]; Richardson [43]; Roberto [44]; Sale [46]; Sale [49]; Sale [48]; Sale [52]
What is a ‘BMD’ test all about?	I don’t understand what a bone mineral density (BMD) test is or what it involves. I don’t understand what the results mean. I can’t make a good decision if I don’t understand what is going on.	Beaton [23]; Lau [33]; Richardson [43]; Sale [45]; Sale [46] Sale [50]
What are the actual benefits of medication?	I don’t know whether or not to take the medication. The side effects might outweigh any benefits. What if it causes cancer? What if the doctor hasn’t told me everything? I find it virtually impossible to follow the necessary procedure. A good diet, exercise and taking care might be enough, but the medication might be the only real option available.	Beaton [23]; Besser [25]; de Souza [26]; Hansen [30]; Iversen [31]; Lau [33] Mazor [34]; Nielson [38]; Paier [40]; Quantock [41]; Roberto [44]; Sale [43]; Sale [41]; Sale [49]; Salter [53]; Solimeo [54]; Weston [55]
Relationship with healthcare professional	I need the doctor to listen to me and to treat me with respect. This means keeping me informed and taking me seriously. Some doctors are too busy or not interested. I know that I should follow the doctor’s instructions, but I also want to be in control of my health. This means asking questions and being involved in healthcare decisions that affect me.	Beaton [23]; Hallrup [24]; Besser [25]; Hallberg [29]; Hansen [30]; Iversen [31]; Lau [33]; Mazor [34]; Meadows [37]; Qvist [42]; Richardson [43]; Sale [49]; Sale [50]; Sale [52]; Weston [55]
Cultural images of the ageing body		
OP synonymous with age and decline	OP is part of growing old and I can’t change this. However, the changes in my body remind me that I am getting older.	Besser [25]; Mazor [34]; Richardson [43]; Sale [50]; Salter [53]; Solimeo [54]; Weston [55]; Wilkins [56, 57]
I am focussing on life’s possibilities	As you get older, you need to focus on enjoying life’s possibilities and taking on new challenges. Getting old is natural and brings the benefit of wisdom. I will try and look on the bright side.	Hallberg [29]; Hansen [30]; Jachna [32]; Nielson [39]; Roberto [44]; Weston [55]; Wilkins [56, 57]
People think osteoporosis is a women’s condition	I sometimes feel embarrassed because people think OP is a women’s condition. I don’t want to go to the doctor and I hide it because I am worried what people will say. I may even lose my job. Men are supposed to be the strong ones.	Nielson [38]; Solimeo [54]
Untranslatable primary data (no gravitational idea)		
	Mckenna [35] and Besser [25]: medication adherence Hallberg [29]: strategies for maintaining independence—activity Jachna [32]: barriers (and benefits) to treatment Lau [33]: barriers and facilitators to adherence Salter [53]: decision making around medication Solimeo [54]: explanatory model—commonality/variability	
Concept not included into conceptual categories		
	French [27]: lifestyle habits and food preferences Quantock [41]: diet and exercise/HRT Qvist [42]: awareness and experiences of the body through back muscle exercise Sale [50]: participants used a variety of non-pharmacological strategies to address fracture risk—diet and supplement use	

## Findings

**Fig. 1:**
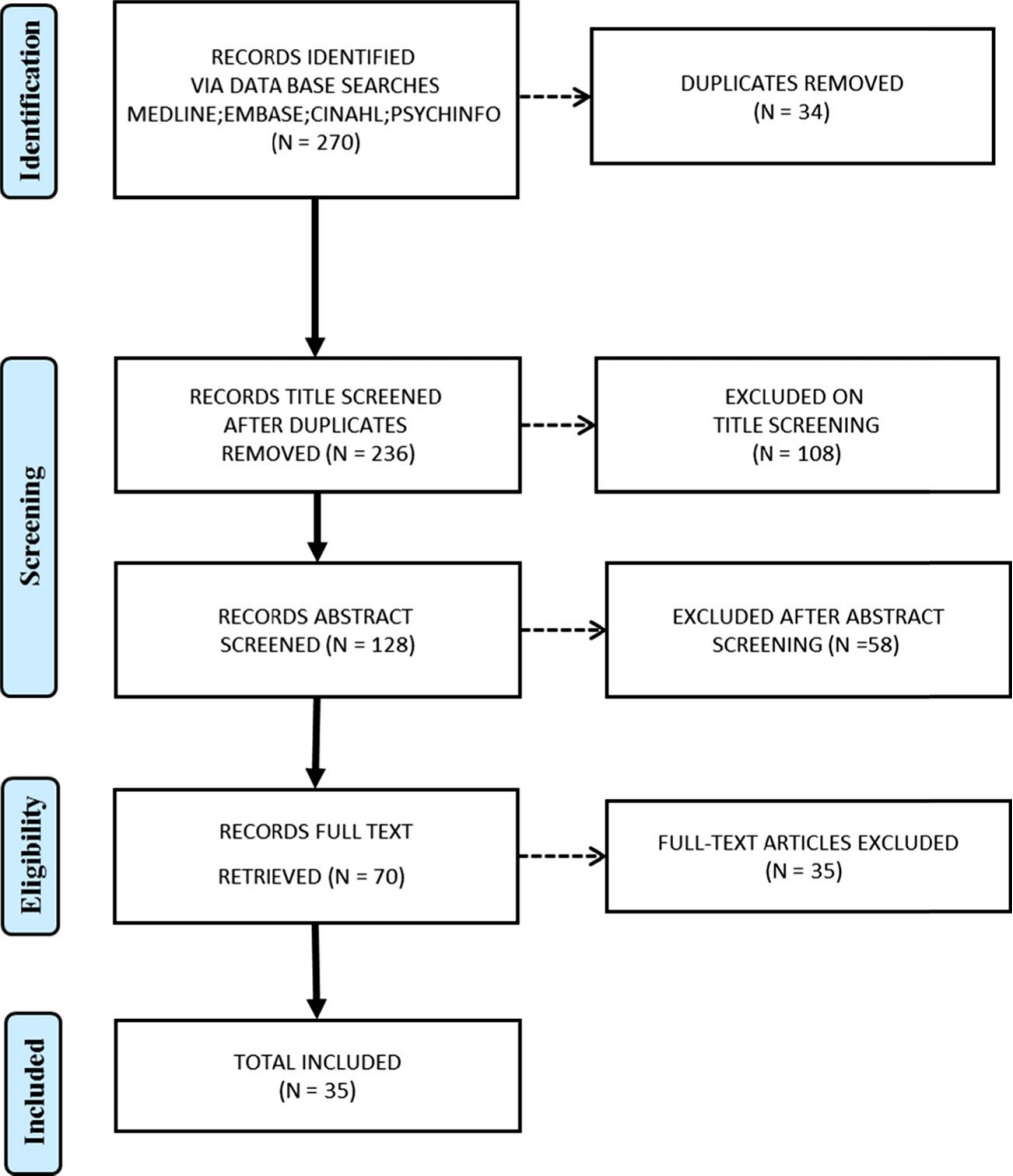
Flow diagram of search

We identified 270 potential qualitative studies (Fig. 1) and removed 34 duplicates. We screened 236 titles, 128 abstracts and 70 full text articles. We excluded 35 from full-text screening that did not meet the inclusion criteria (for example there was no diagnosis of osteopenia or OP). We included 35 papers that reported 34 unique international studies: Canada (*n* = 14), USA (*n* = 6), UK (*n* = 6), Sweden (*n* = 3), Denmark (*n* = 3), Brazil (*n* = 1) and UK and Denmark (*n* = 1). These qualitative studies explore the experience of 773 participants, of which 83 were men (Table 3) [23–56].

**Table 3:** Included studies

	Age reported	Country	Sample context	Number	Data collection analysis
Beaton [23]	64 (47–80)	Canada	Fragility fracture: fracture clinic	24 (18 women)	5 focus groups, grounded theory
Hallrup [24]	76–86	Sweden	Fragility fracture: fracture prevention programme	13 women	In-depth interview, phenomenology
Besser [25]	69 (SD 10.1)	UK	OP/osteopenia and medication: OP screening unit/rheumatology	14 women	Semi-structured interviews, thematic analysis
De-souza [26]	36–79	Brazil	OP; calcium disorders unit	12 (11 women)	Non-structured interviews grounded theory
French [27]	67.4 (52–87)	Canada	Osteopenia (13)/OP (17): OP treatment programme	30 women	Focus group, thematic analysis
Giangregorio [28]	67.5 (SD 12.7)	Canada	Fragility fracture: fracture clinic	127 women	Telephone interview, thematic analysis
Hallberg [29]	68–84	Sweden	Fragility fracture (vertebral): follow-up study	10 women	Semi-structured interviews, thematic analysis
Hansen [30]	65–79	Denmark	Fragility fracture (first known): DEXA scan clinic	15 women	Open interviews, phenomenology
Iversen [31]	65–85	USA	OP/osteopenia and medication: adverts in hospital newsletter	32 (30 women)	3 focus groups, thematic analysis
Jachna [32]	84 (71–93)	USA	OP and fragility fracture (hip): assisted living facility	5 women	Semi-structured interviews, content analysis
Lau [33]	70 (48–88)	Canada	OP/osteopenia and medication: primary and secondary healthcare professionals	37 women	7 focus groups, phenomenology
Mazor [34]	73.4 (SD 6.2)	USA	OP/osteopenia and medication/multi-specialty group practice	36 women	Telephone interview, thematic analysis
Mckenna [35]	43–82	UK	OP: support groups; exercise classes and 5 Asian community centres.	21 women	Semi-structured interviews, phenomenology
Meadows [36]	40–65	Canada	Fragility fracture: women and bone health study	19 women	Semi-structured interviews, thematic analysis
Meadows [37]	40–65	Canada	Fragility fracture: women and bone health study	24 women	4 focus group, thematic analysis
Nielson [38]	51–82	Denmark	OP (men): department of endocrinology	16 men	4 focus groups, phenomenology
Nielson [39]	50–84	UK/Denmark	OP and DEXA scan: OP management or self-help group	14 (10 women)	Semi-structured interviews, phenomenology
Paier [40]		USA	Fragility fracture (vertebral): via healthcare professionals	5 women	Semi-structured interviews, phenomenology
Quantock [41]	70 (65–76)	UK	OP (severe): OP service	11 women	Focus group, thematic analysis
Qvist [42]	68 (60–93)	Sweden	Fragility fracture (vertebral): exercise programme	11 women	Open interviews, thematic analysis
Richardson [43]	33–81	UK	DEXA scan: DEXA scan clinic	15 women	Semi-structured interviews ‘template analysis’
Roberto [44]	53–89	USA	OP: community adverts	21 women	4 focus groups, thematic analysis
Sale [45]	49–82	Canada	OP: OP-screening programme	18 (14 women)	Semi-structured interviews, phenomenology
Sale [46]	47–80	Canada	Fragility fracture and DEXA scan: OP-screening programme	24 (18 women)	5 focus groups, thematic analysis
Sale [47]	65–88	Canada	Fragility fracture: OP-screening programme	30 (21 women)	Semi-structured interviews, phenomenology
Sale [48]	50–79	Canada	Fragility fracture: OP-screening programme	25 (22 women)	Semi-structured interviews, phenomenology
Sale [49]	65–88	Canada	Fragility fracture: OP-screening programme	21 (15 women)	Semi-structured interviews, phenomenology
Sale [50]					
Sale [51]	51–89	Canada	Fragility fracture: advert in patient newsletter	28 (26 females)	Telephone interview, phenomenology
Sale [52]					
Salter [53]	70–85	UK	OP and medication: multi-centre trial	30 women	Semi-structured interviews, thematic analysis
Solimeo [54]	70.36 (53–86)	USA	OP (men): bone health clinic	23 men	Semi-structured interviews, thematic analysis
Weston [55]	68–79	UK	OP and medication: OP screening trial	10 women	Semi-structured interviews, phenomenology
Wilkins [56, 57]	65.3 (54–80)	Canada	OP: OP clinic or self-help group	28 women	In-depth interview, thematic analysis

We appraised all of the articles using the CASP quality appraisal checklist [21]. There was considerable discrepancy in scoring between quality reviewers. There were only 2 studies where both reviewers agreed on the quality score, and 10 studies had a discrepancy of at least 10 %. The correlation between the sets of scores was 0.54, i.e. only moderate agreement.

The conceptual categories and supporting studies are shown in Table 2. Six papers included sections where FT and CML were unable to decipher a coherent concept; six concepts were not included in the conceptual analysis, as they did not represent a gravitational idea. These are also shown in Table 2. For transparency, we have listed concepts that we did not think fit the conceptual categories in order to allow the reader to consider the placement of these concepts (Table 2). Readers may feel that these concepts fit under the umbrella of our suggested categories or that they contribute to additional categories to be considered.

## Negotiating the visibility and invisibility of osteoporosis

Central to the qualitative findings in this review is the person’s struggle to negotiate the visibility or invisibility of OP. Participants discovered their OP in various ways. For example some found out by chance through diagnostic screening following a fracture or as a result of routine tests for coexisting health condition. Some initiated their own testing as a result of pain or physical changes such as loss of height. Coming to terms with this diagnosis was a process linked to the relative visibility or invisibility. Whilst some accepted that the symptoms were linked to their bone health (and made adaptations), others did not make this link. Some preferred not to find out as ‘knowing [gives me] something to worry about’.

Our first overriding conceptual category describes OP as *not* manifest. In this category, a person’s personal narrative, or biography, which describes who they are, remains intact: I know I have got it but I can’t see it; there was nothing fragile about my fracture; I am not the type to get OP; it is not as bad as other conditions. Our second category describes OP as manifest and personal biography as fractured: OP choreographs my life; I am becoming isolated; I don’t want to rely on other people; living in fear of falls and fractures; fear of what is to come; I am watching my body get old. Our third conceptual category describes an overwhelming uncertainty that pervades the experience of OP: what is my risk? What is a bone mineral density (BMD) scan all about? What are the actual benefits of medication? This uncertainty hinges on the patients relationship with their healthcare professional which is integral to the process of determining risk and decision making. Our final theme demonstrates how the experience of OP is set within a cultural context with certain views about ageing and gender.

## Biographical integrity—osteoporosis is not manifest

This category includes conceptual themes that describe OP as not manifest and personal biography as intact.

### I know I have got it but I can’t see it

This describes OP as occupying an invisible place in everyday life. Some remain able to accommodate the disease process and retain their biographical integrity. Some struggled to accept a diagnosis of OP because they felt healthy and had no visible signs. There was a sense that visible symptoms (e.g. pain) would be a warning sign of damage and potential risk (‘you must be able to feel something’). Even those who accepted the diagnosis might choose to stop or relax treatment at times that the disease was not manifest (‘that’s the hard part because you can’t see anything’).

### There was nothing fragile about it

Participants did not always link bone fragility and fracture. Some described the circumstances of their fracture as ‘traumatic’ and gave vivid descriptions of traumatic events. At times, this view was supported by the HCP; ‘[he said that] anyone would have fractured in these circumstances’. Some were shocked because their fracture followed an innocuous event.

### I am not the type to get OP

Even when participants understood the link between bone health and fracture, they did not always feel that they were *personally* at risk. Some were shocked if the scan was positive. Some felt that because they had lived a healthy life with a good diet and plenty of exercise, this protected them from developing poor bone health. Others felt that they were protected by physical attributes, strong genetic makeup or that they were just too young (‘I always had good strong bones as far as I know’).

### It is not as bad as other conditions

OP could retain relative invisibility because participants prioritised other health concerns (such as heart disease or diabetes), particularly if symptoms of OP were not manifest. Some compared themselves to people with other ‘more serious’ conditions such as dementia or cancer. Some did not regard OP as serious even following a fracture or when taking OP medication (‘I mean, I could have lung cancer or dementia. … I think I’m lucky’).

## Biographical fracture—osteoporosis is manifest

This category includes conceptual themes that describe OP as manifest. In this category, a person’s personal biography has been fractured, specifically individuals who are experiencing mobility challenges and/or pain from fractures.

### OP choreographs my life

This describes the biographical disruption of OP fracture. OP could have a profound impact on mobility, work and social lives. Some described deep emotions such as shock, anger, sadness and fear. For some, the role of pain in choreographing daily activities could continue long after fracture repair.

### I am becoming isolated

Loving and caring relationships were felt integral to health and quality of life. Some had become isolated at home or dependent on family and friends for social contact. Continuing pain could also affect relationships with family and friends. For example some avoided social situations. Others described feelings of vulnerability, loneliness and abandonment. Sharing experiences about OP with other people who had OP could foster experiences of affinity and increase confidence.

### I don’t want to rely on other people

Personal autonomy and independence were also described as integral to good health and quality of life. Dependency on family members profoundly altered established social roles. Having to accept help (particularly with personal care) was one of the most difficult things to do. At times, relatives could be ‘too’ helpful, but equally, it was not easy to ask for help when it was needed. Although pleased to receive help, this was not always available and this change in role could become a frank reminder of the process of getting old.

### Living in fear of falls

This describes vigilance about living in a world that is now viewed as dangerous. Some felt deeply threatened by activities that would not normally pose a threat. Some managed their risk of fractures by taking great care to prevent falls, rather than through diet, exercise or medication. Aids and devices become one way of controlling risk. Caution became a natural habit and could contribute to social isolation as people chose to stay at home in a ‘safe’ environment.

### Fear of what is to come

This describes deep concern with what the future might bring. Hope hinged on success of treatment or being able to successfully accommodate manifestations of OP and was countered by fear of unpredictable consequences. Participants described fears of losing mobility, of being wheelchair bound, of being dependent on others and of further fractures, falls and deformity.

### I am watching my body get old

The physical manifestations of OP were described as synonymous with becoming ‘old’. Loss of height and spinal deformity were described as the hallmark of both OP and ageing. At times, this was underpinned by negative cultural meanings of ageing. Some were reluctant to accept the diagnosis of OP because they saw the physical changes as a mark of being old which threatened personal identity. Participants spoke profoundly of personal diminishment (I am shrunken, stooped, bent). Some were haunted by the spectre of someone who embodied this image for them; ‘I couldn’t believe it … this woman in such marvellous shape … all of a sudden here she is with this … debilitating thing’.

## Overwhelming uncertainty

This conceptual category describes an overwhelming uncertainty that pervades the experience of OP.

### What is my risk?

This describes overwhelming uncertainty about fracture risk. There was an underlying sense that ‘everyone gets a bit of OP’ which could downplay a sense of risk. Some had never received or discussed their BMD tests with a healthcare professional. Some assumed that no news was good news, even if they were prescribed OP medication. Participants described confusion and worry about inconsistent information. Some remembered being given inaccurate advice such as the following: you are protected by your physical make-up and don’t need testing, or, older people should take medication to prevent OP even when they don’t have OP. Some actively sought out other sources of information, for example other health professionals, written material, friends, family or other people with OP.

### What is a ‘BMD’ test all about?

This describes uncertainty about the meaning and process of BMD testing confusion over risk status. Some felt it would be an invasive test and were pleasantly surprised. A good understanding of test results could help participants to evaluate their risk and decide what to do.

### What are the actual benefits of medication?

This describes the complex process of deciding whether or not to take medication to manage OP. This process could be straightforward or accompanied by worrisome thoughts and the decision could alter depending on the context. For some, their default decision was *not* to take medication as the side effects outweighed the manifestations of OP. Participants described fears and suspicions of medication (for example will it cause cancer? have I been fully informed? Is the doctor over-prescribing? What is the agenda of pharmaceutical companies?) The complexity of administering the medication and a lack of understanding of what it was doing to you could also be a disincentive. Some personally disliked the idea of taking medication and preferred to make lifestyle modifications, whereas for others, medication provided a feeling of safety and was regarded the only solution to prevent decline.

### Relationship with healthcare professional

The relationship between healthcare professional and patient was described as integral to negotiating uncertainty. This relationship could help or hinder the processes of determining risk and deciding how to manage OP. A therapeutic relationship incorporated the following: being listened to, being treated with respect, being kept informed and being taken seriously. Some described the doctor as too busy or as ‘not interested’. For some, the patient’s role was to follow the doctor’s instructions. This could be vested in previous good experience or a paternal view of healthcare provision (‘I know the doctors can’t be wrong’). Others described the patient’s role as self-advocate for their own health. This involved taking control of your own health by seeking information, asking questions and actively seeking specialist referrals or specific medications (‘we had to wheedle and deal around that a long time before he finally agreed’).

## Cultural images

Our final conceptual category demonstrates how the experience of OP is set within a culturally specific context that incorporates cultural constructs of the ageing body and OP as a women’s condition.

## The ageing body

### OP synonymous with age and decline

Participants described the inevitability of OP as a normal part of ageing that is beyond personal control (‘… the crumbly status of old age’). Some talked about their fracture risk in terms of physical instability that comes with old age (‘becoming a bit doddery’) rather than bone fragility. Others described OP as a chronic lifelong condition of ageing where bones have become ‘weak’ or ‘brittle’ or ‘thin’, or confused it with ‘wear and tear’ or arthritis. The physical manifestations of OP (I can see my body getting old) could be a frank reminder of age and decline.

### I am focussing on life’s possibilities

This describes positive cultural images of the ageing body. For example some describe the need to focus on enjoying the possibilities of older adulthood and taking on new challenges. Maintaining meaningful and valued occupations was described as integral to good health, quality of life and a positive sense of self. Some regarded ageing as a natural process, even a time of increased wisdom that brought change and potential benefit; ‘I’m really proud of myself of being a new member of the rowing club’. Personal resources and a positive approach to life were viewed as protective, whereas negative thinking and worry could negatively affect a life of health.

## Gender—osteoporosis is a women’s condition

One study [26] described how the female gendering of OP could have a profound effect on men’s experience of living with OP and their decision to seek help. Some described the shame and embarrassment of living with a ‘female disease’ and chose to hide their diagnosis for fear of ridicule (or even job loss). Men referred to the cultural construct of men as strong and described their incapacity to live up to this construct; ‘I accepted that I am a sissy a long time ago’. Some also perceived the need for health care as weakness and, therefore, as not masculine; ‘If osteoporosis wasn’t looked upon as a female disease, more men would seek help’.

## Conceptual model

**Fig. 2:**
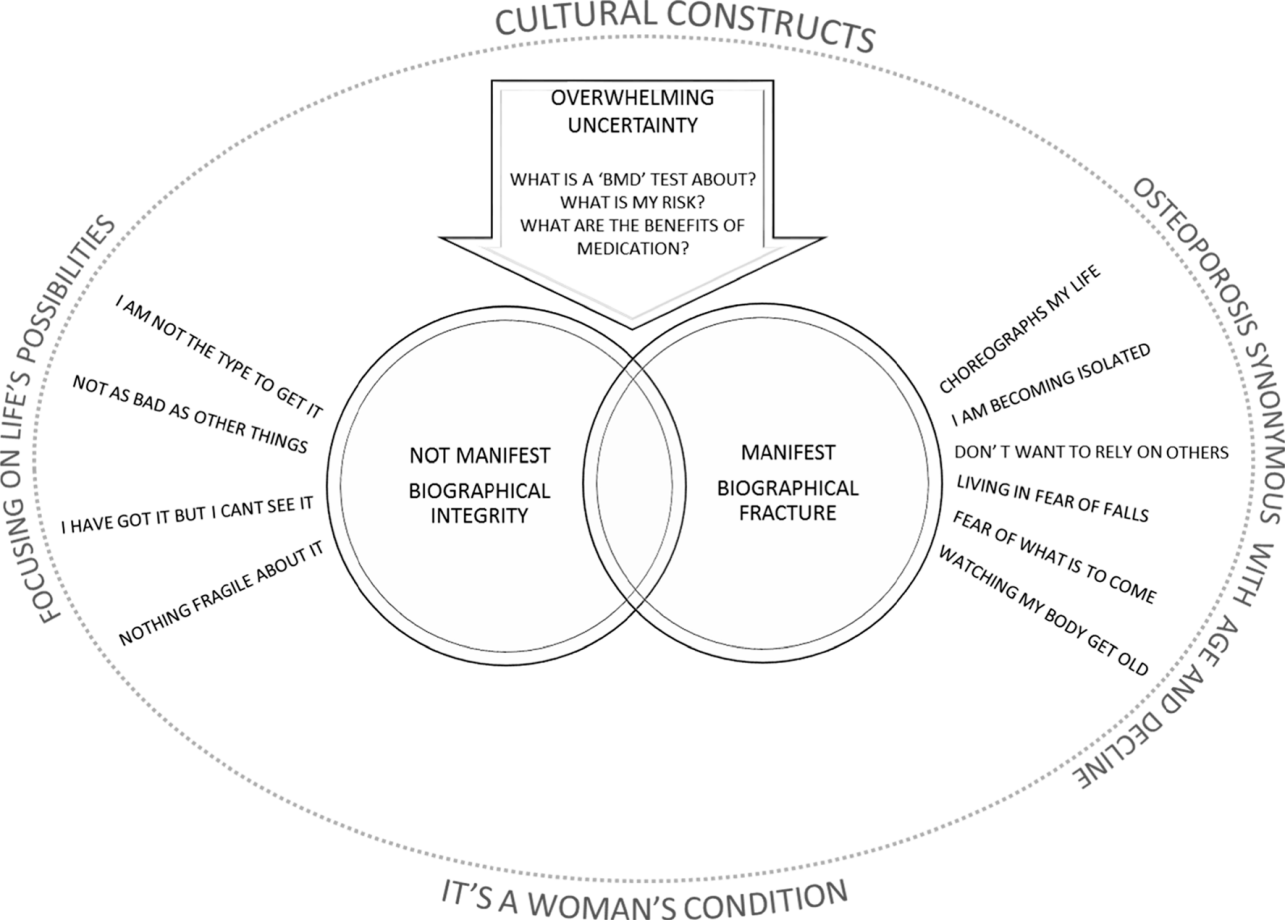
Conceptual model

Our conceptual model hinges on the negotiation between invisibility and visibility of osteoporotic symptoms. On the one side, personal biography remains intact (I am not the type to have OP, I can’t see it; it is not as bad as other things and there was nothing fragile about my fracture). On the other side, personal biography is fractured (it choreographs my life; I am becoming isolated; I don’t want to rely on others; I am living in fear of falls and what might come; I am watching my body become old). Self-construct hinges upon negotiating this balance between integrity/fracture of personal biography and visibility/invisibility of symptoms. Our model draws attention to the relationship between *fractured body* and a *fractured sense of self*. Negotiating visibility/invisibility of OP is accompanied by an overwhelming uncertainty (what is a BMD test; what is my risk; what are the benefits of medication?) which can be influenced, both positively and negatively, by a person’s relationship with their healthcare provider. This experience of living with OP is set within a cultural framework with certain views about ageing and gender. On the one hand, the physical manifestations of OP are seen as synonymous with age and decline, yet at the same time, focussing on life’s possibilities in older adulthood provides a cultural image of ageing well. Gendered views of OP as a women’s condition provide another cultural construct that influence the experience of OP (Fig. 2).

## Discussion

This is the first international study to systematically review and integrate qualitative research in order to increase our understanding of people’s experience of what it is like to live with OP. The review suggests that patients navigate living with this chronic condition in diverse ways. Our findings resonate with those found in the ‘Life with Osteoporosis project’ conducted by the UK National Osteoporosis Society [58]. In this project, 3228 people completed a questionnaire about living with OP, and 27 people took part in in-depth interviews. This survey support the wide-ranging impact of OP on people’s lives, particularly, giving up the things you love; emotional insecurity and fear of ridicule because of changing body shape; fear of losing independence; and not being able retain physical contact with loved ones. The survey found that 30 % of people found OP a financial burden, and a quarter of people who were working at the time of diagnosis had given up their job or made changes to their working lives.

Central to the qualitative findings in this review is the person’s struggle to negotiate the visibility (manifest) or invisibility (not manifest) of OP. People with OP negotiate a balance between invisibility and visibility of osteoporotic symptoms, and self-construct can hinge upon successfully negotiating this. The study participants describe how they struggled to understand and give meaning to their symptoms [55, 59, 60].

The review sought to include papers that gave insight into the experience of living with a diagnosis of osteoporosis. However, participants included were those with osteopenia and those with a history of multiple fragility fractures but where the original authors had not been explicit that the respondents met the definition of osteoporosis based on *t*-score of −2.5 SD on DEXA scan. All participants, however, clearly had significant poor bone health and a high risk of fragility fracture and as such, we believe that there is commonality in the issues raised by them irrespective of whether their *t*-score categorised them as osteoporotic or osteopenic.

The review demonstrates contrasting feeling; on the one hand, OP is invisible and fragility fractures do not accord with the lived experience of symptoms that they could observe or feel; conversely, others interpreted the diagnosis as inhabiting a body that could be easily damaged with little or no provocation. The process can be accompanied by overwhelming uncertainty. We see how patients might not fully understand tests, risk or how to decide what action to take. This overwhelming uncertainty is underpinned by a person’s relationship with their healthcare provider. The lack of understanding is important as without a clear understanding about the potential health impact and the importance of adherence to both pharmacological and non-pharmacological strategies aimed at bone health, outcomes may be adversely affected [23, 27, 31, 33]. The experience of living with OP is set within a cultural framework with certain views about ageing and gender. On the one hand, the physical manifestations of OP are seen as synonymous with age and decline, yet at the same time, focussing on life’s possibilities in older adulthood provides a cultural image of ageing well. Many of the studies reported OP as a natural progression expected with age, suggesting that patients might made sense of their diagnosis through a fatalistic acceptance [30]. Gendered views of OP as a women’s condition provide another cultural construct that influence the experience of OP.

There are methodological issues to be considered for qualitative syntheses [61]. For example how many studies should be included? Noblit and Hare do not advocate an exhaustive search [13], and the number of studies included in meta-ethnography ranges widely [10, 18]. Meta-ethnography does not aim to summarise the entire body of knowledge, or make statistical inference, but focusses on conceptual insight. We did not exclude studies as a result of methodological appraisal, and this is not an uncommon decision for qualitative syntheses. Inter-rater reliability for qualitative appraisal tools is low and does not necessarily have bearing on a studies conceptual insight. There is no consensus on what makes a qualitative study ‘good’ or ‘good enough’ and not agreed framework for doing this [10, 18]. Although appraisal tools are often used in qualitative synthesis [21], the majority of qualitative syntheses (27 out of 41) identified by Campbell and colleagues’ did not use appraisal criteria to determine inclusion [10]. Where tools are used to appraise the quality of qualitative research, there tends to be low agreement between researchers [18]. Our findings support this. However, these checklists are useful in providing a focus for discussions [10]. Some argue that quality appraisal should not be used at all to exclude studies from qualitative synthesis [61]. As appraisal tools tend to focus on method, some argue that excluding studies on this basis may mean that insightful conceptual studies are excluded [10]. Although some experts suggest that studies should not be excluded on the grounds of quality, they do not recommend ‘abandoning appraisal’ altogether [10]. Conceptual insight is fundamental to meta-ethnography, and therefore, inclusion is determined by clarity of ideas.

We have used established methods [13, 61, 62] to develop a conceptual model that helps us to understand what it is like to live with OP. We have included studies where participants have a diagnosis of OP or osteopenia and fragility fracture. It might be that the experience of OP is different for those with fractures at different sites (e.g. hip or vertebral fracture) or for different ages or genders. Research including homogenous groups of participants with OP could add insight.

A number of issues are identified that are pertinent to clinical practice, especially the importance of giving the diagnosis of OP. It is also vital that healthcare professionals check that the patient understands their diagnosis as the themes of uncertainty and invisibility clearly show that patients’ struggle to understand the meaning and the implications of this diagnosis. The theme that OP is set within a patient’s cultural framework is important for clinicians to understand, particularly in relation to adherence to advice regarding diet, exercise and medication. This is particularly pertinent to men, who feel that OP is a female disease, despite the statistical likelihood that it will affect 20 % of them. Similarly, we need to be cognisant that all communication needs to be culturally sensitive for ethnic groups who are known to be at greater risk due to low vitamin D and greater levels of social isolation [63, 64]. Overall, our synthesis has highlighted the wealth of qualitative data about OP. Despite the increasing body of literature on the subject, there remains a need to adjust our interactions with patients to understand how patients can be helped to receive and understand their diagnosis and move forward in partnership with healthcare providers to promote optimal management of the disease.
